# Gait in childhood and adulthood in persons with myelomeningocele – a retrospective analysis

**DOI:** 10.1186/s12883-026-05094-y

**Published:** 2026-06-23

**Authors:** Marie Eriksson, Elena M Gutierrez-Farewik, Åsa Bartonek

**Affiliations:** 1 Department of Women’s and Children’s Health, Division of Paediatric Neurology, Karolinska Institutet, Karolinska University Hospital, Motoriklab QA: 27, Karolinska vägen 37, Stockholm, S-17176 Sweden; 2 Department of Engineering Mechanics, KTH MoveAbility, KTH Royal Institute of Technology, Stockholm, Sweden

**Keywords:** Spina bifida, Meningomyelocele, Ambulation, Orthotics, Kinematics, Kinetics, Joint work

## Abstract

**Background:**

Gait patterns in children with myelomeningocele (MMC) at various neurological levels have been described, both with and without orthotic support. Although the neurological level of the lesion serves as an important predictor of ambulatory potential, the expected walking ability is not always achieved, as additional factors such as spasticity may influence gait negatively.

The aim of this study was to retrospectively compare gait patterns as assessed in childhood with those observed in adulthood.

**Methods:**

Of 59 individuals with MMC aged 18 years or older, 29 had undergone three-dimensional gait analysis in childhood (Ch-GA). These data were retrospectively analysed and compared with findings from a subsequent adult gait analysis (Ad-GA). The mean (standard deviation) age at the time of Ch-GA was 11.6 (4.1) years and at Ad-GA 25.9 (3.9) years. The median (range) interval between assessments was 15.0 years (5.1–17.2).

**Results:**

Twenty-two participants maintained independent, non-assisted walking (Group A), 5 had transitioned from independent walking to using a walking aid (Group B), and 2 used a walking aid at both Ch-GA and Ad-GA (Group C), with individualized orthotic prescriptions provided at both time points. In Group A, two of eleven kinematic variables and six of eleven kinetic variables in the hip, knee, and ankle showed deterioration, and walking speed had decreased. Functional ambulation declined from 18 community ambulators and 4 household ambulators (Ha) in childhood to 8 and 14, respectively, in adulthood. In Group B, analysed with only Gait Deviation Index (GDI), values were unchanged, but all temporospatial gait parameters had deteriorated. Functional ambulation decreased from five individuals classified as Ha to two Ha and three non-functional ambulators. The two individuals in Group C, who used a walker at both assessments, largely maintained the same GDI values and temporospatial parameters as in childhood.

**Conclusion:**

Largely consistent with our original expectations, the findings indicate that gait patterns remain relatively stable from childhood to adulthood in individuals with MMC when supported by appropriate rehabilitation interventions, though some deterioration of gait and ambulation occurred. The results reflect gait-related changes that can be expected during growth in this population.

**Supplementary Information:**

The online version contains supplementary material available at 10.1186/s12883-026-05094-y.

## Background

Myelomeningocele (MMC) is the most common form of spina bifida (SB), occurring in approximately 1 per 1,000 births worldwide. A baby with MMC is born with a malformation of the spinal cord, characterized by an incomplete dorsal closure of the vertebrae, often presenting with urinary and faecal incontinence and hindbrain herniation-associated hydrocephalus. Neurological deficits below the level of the lesion may be present, involving both motor and sensory functions, resulting in lower-limb paresis that hinders or prevents walking. A strong correlation has been demonstrated between the axial lesion level and the degree of disability experienced by patients [1].

The use of orthoses during walking plays a crucial role in stabilizing joints that become unstable due to muscle paresis. This requires orthotic treatment using a variety of models adapted to each individual’s specific conditions [2]. Gait has been studied using three-dimensional (3D) gait analysis in children with SB and MMC. In children with lumbar and sacral neurological levels, Duffy et al. identified recognisable gait patterns in barefoot walking specific to each SB level, with deviations accurately reflecting the underlying muscle deficiencies. The most important findings were persistent knee flexion throughout stance, along with increased pelvic obliquity and rotation, and hip abduction during stance [3]. 

Compared with barefoot walking, ankle-foot orthoses (AFOs) have been shown to significantly improve sagittal plane kinematics and kinetics in persons with low-level MMC [4]. In a group of persons with MMC who wore AFOs, the use of crutches resulted in higher stride length and lower cadence compared to walking without crutches, although walking velocity did not differ [5]. In a study of gait kinematics and energy expenditure in independent ambulators with MMC, exaggerated pelvic obliquity demonstrated the strongest association with oxygen cost, suggesting that hip abductor strength plays a key role in the energy demands of gait [6].

We have previously reported in studies using 3D movement analysis that upper body segment motion increases with increasing degree of lower-limb muscle weakness [7], and have identified characteristic gait kinematics [8], kinetics [9] and temporospatial parameters in children with different levels of weakness in lower limb muscles. Hip abductor paresis was identified as the most influential muscle deficit affecting gait kinetics. In these studies, children with extensive hip extensor and abductor weakness used a knee–ankle–foot orthosis (KAFO) that freely articulated in flexion/extension to stabilize the knee in the transverse and frontal planes, accommodating compensatory upper-body motion and preserved self-ambulation. The use of AFOs in children with MMC is frequently recommended to prevent excessive dorsiflexion during stance and to increase walking velocity, although caregivers have been advised to monitor for potentially increased transverse rotational forces at the knee [10]. In independently walking children with low-level MMC and plantarflexor weakness, we compared walking in regular AFOs and KAFOs vs. orthoses equipped with an energy-restoring carbon fibre spring ankle component and found that the carbon fiber component resulted in higher ankle plantarflexion moment, ankle positive work, and stride length [11]. Likewise, Wolf et al. reported that use of a carbon fibre spring significantly increased energy return during the third rocker, more closely simulating natural push-off [12].

In a small group of children with mid-to-high lumbar and thoracic lesions, gait training with an isocentric reciprocating gait orthosis was reported to increase walking speed, step length, and hip sagittal plane range of motion, and to decrease vertical and horizontal compensatory motions [13].

While numerous studies have examined gait in 3D in children, relatively few have focused on adults with MMC. In a simple model with three reflective markers on the malleoli and shoes in adults over 50 years with spina bifida, walking speed was negatively influenced by SB severity [14]. In a study using wearable sensors on the upper and lower body on adults between 18 and 65 with MMC during walking with their regular orthoses or walking aids, persons with less muscle function showed higher lateral sway in the lumbar and thoracic region than those with lower lesion level [15].

While the neurological lesion level is an important predictor of walking potential, expected walking ability is not always achieved due to factors such as age, presence or absence of hydrocephalus, spasticity, spinal deformities, cognitive impairment, lower-limb fractures, contractures, and social conditions [16]. Factors associated with deteriorated ambulation were found to be number of shunt revisions, balance disturbances, and spasticity of the hip and knee joints [17]. Spasticity has been reported to primarily affect muscles below the neurological lesion level, further impairing gait [18] and possibly necessitating greater orthotic support than would be indicated by motor paresis alone [17, 19]. These additional factors have been shown to impair walking ability already by the age of 6 years [20].

To establish realistic goals for ambulation, Hoffer et al. [16] introduced four functional levels of ambulatory ability in children (community ambulators, household ambulators, non-functional ambulators and non-ambulators). Including assessment of arm function has been proposed, as it may be affected by Chiari II malformation in children with MMC [21]. 

Recently, studies have reported that in children with MMC, functional mobility and motor levels may improve in those with prenatal versus standard postnatal repair [22].

Although orthosis use is common in individuals with SB, evidence supporting the efficacy of orthotic management in ambulant children and adolescents remains limited [23].

Since the early 1990s, active orthotic management for individuals with MMC has been available through a single orthotic clinic working in collaboration with the multidisciplinary team at Karolinska University Hospital [2]. This concept, inspired from a group in Italy, incorporates expected gait function, biomechanical principles, orthopaedic and neurological status, and orthosis timing [24]. Orthotic guidelines and recommendations for the timing of orthoses and physiotherapy during childhood have been reported earlier in Swedish [20] and German populations [25]. In short, these are that besides parent information, the local physiotherapists were instructed to pay attention to each child’s motor initiatives toward a vertical position and to offer appropriately-timed orthoses to promote standing and walking.

The children and adolescents with MMC who participated in our studies from the 2000s described above have undergone this active orthotic concept during childhood signs [7–9, 11, 17, 19, 20]. In recent studies, we described that the orthosis use in the same population from childhood to adulthood has changed only minimally across age groups [2], and that orthotic management has remained highly individualized [26]. To our knowledge, no studies have reported 3D gait analysis parameters in adults who had also previously been studied as children. The aim of this study was therefore to compare the gait patterns in individuals who participated in the previous studies in the late 1990s and early 2000s described above [7–9, 11, 19] with their gait patterns in the late 2010s as adults. We hypothesized that gait patterns observed in childhood would remain largely similar to those observed during adulthood.

## Methods

### Participants

Fifty-nine persons with MMC, aged 18 years or older and born in or after 1985, took part in a study between February 2018 and June 2019 of physical function, orthosis use, and ambulation [2, 26]. Of this group, 30 were available for a gait analysis, of whom 29 had previously undergone a 3D gait analysis as children (Ch-GA) at the same gait laboratory; thus, 29 persons were included in this study. Mean (standard deviation, SD) age at Ch-GA was 11.6 (4.1) years, and at adult GA (Ad-GA) was 25.9 (3.9) years. The interval between Ch-GA and Ad-GA was median (min, max) 15.0 (5.1, 17.2) years. The study was approved by the Regional Ethical Review Board in Stockholm, Dnr 2017/910 − 31/4 and written informed consent was obtained from all participants.

At Ad-GA, all participants underwent a clinical examination by the same examiner as in childhood (ÅB). Based on strength testing of lower limb muscles using a 0–5 graded scale [27], participants were assigned to a muscle function class (MFC) [17]. Participants with weakness in foot intrinsic muscles and plantarflexors graded 4–5 were classified as MFC I; those with foot plantarflexion grade 3, knee flexion grade 3, and hip extension and/or hip abduction graded 2–3 were classified as MFC II; and those with hip flexion and knee extension graded 4–5, knee flexion grade 3, and only traces of hip extension, hip abduction, and below-knee muscles were classified as MFC III. Passive joint range of motion in the hips, knees, and ankles was measured with a goniometer in the supine position. Hip flexion contracture was assessed using the Thomas test [28]. A joint contracture was defined as a reduced range of motion compared to the neutral position. Spasticity in the lower limb muscles was documented, as well as additional neurological signs, including balance disturbances during standing and walking (in the presence of quadriceps strength) despite the use of adequate orthoses; excessive fear when sitting upright without back support (in the presence of hip flexor strength); and generalized muscle hypotonia not attributable to flaccid paresis of individual muscle groups [17].

Orthosis types and functional ambulation were documented at Ad-GA and retrospectively at Ch-GA from the medical chart in the Prosthetic and Orthotic clinic (PO-clinic). Orthosis types were grouped in accordance with the international classification [29] as foot orthosis (FO), AFO and KAFO. A modification was made by specifying KAFO-F (free articulation of knee flexion/extension) and KAFO-L (locked knee flexion/extension).

Functional ambulation was recorded using a four-level scale according to Hoffer: participants walking outdoors were classified as community ambulators (Ca), those walking indoors as household ambulators (Ha), and those walking only for a limited time at home as non-functional ambulators (N-f) [16].

Orthopaedic surgical procedures involving the lower limbs and spine, as well as neurosurgical procedures performed between Ch-GA and Ad-GA, were obtained from the participants’ medical records.

Participants were asked to report pain in the lower limbs, pelvis, and back on the day of examination on a visual analogue scale (VAS) [30].

### Gait analysis

At both Ch-GA and Ad-GA, participants were assessed using a three-dimensional motion analysis system with eight cameras (Vicon^®^ MX40, Oxford, UK) operating at a sampling rate of 100 Hz. A conventional full-body biomechanical model (Plug-In-Gait), based on the Newington model [31], with 34 reflective markers attached to anatomical landmarks on the head, trunk, pelvis, and lower limbs was used. Markers were placed on the orthoses as close as possible to the corresponding anatomical joints and body segments.

Data were collected while participants walked at a self-selected velocity along a 10-meter walkway equipped with two embedded force platforms (Kistler^®^, Winterthur, Switzerland). Gait analyses were performed with each participant’s habitual orthoses and shoes, or with shoes only.

### Data analysis

A minimum of three walking trials per participant was collected. Three-dimensional motion analysis data were processed in Vicon Nexus software. Raw marker trajectories were filtered using a Woltring filter. Kinematic, kinetic, and temporospatial parameters were averaged across available trials. The following kinematic parameters were analysed: average trunk tilt, range of lateral trunk sway, range of trunk rotation, average pelvic tilt, maximum pelvic obliquity in swing, range of pelvic rotation, maximum hip extension, range of hip flexion–extension, maximum knee flexion in swing, maximum knee extension at mid-stance and average external foot progression angle in stance. Internal joint moments at the hip, knee, and ankle were normalized to body weight. The following internal joint moments were analysed: maximum hip extension moment, average hip abduction moment in stance, minimum knee extension moment at mid-stance, average knee valgus moment in stance, maximum dorsiflexion and plantarflexion moment. Joint work was calculated as the time integral of joint power and presented as contribution (as percent) of total positive work from the hip, knee, and ankle. The Gait Deviation Index (GDI), summarizing nine lower-body kinematic variables into a multivariate measure of overall gait deviation, was calculated. A GDI of 100 approximately represents typical gait kinematics, and each 10-point reduction corresponds to one standard deviation from normal [32]. GDI at Ch-GA and Ad-GA was computed from the lab’s reference database of 37 typically developing children and 81 non-disabled adults, respectively. Temporospatial parameters were non-dimensionalized (N) according to Hof [33] and are presented as N stride length, N step length, and N walking speed. All gait parameters are reported as the mean of the left and right sides.

### Statistical analysis

Descriptive statistics are presented as mean and SD, median, minimum (min), and maximum (max) values, based on data distribution. Wilcoxon Signed Ranks tests were used to evaluate differences in GDI between the left and right legs and to compare GDI, kinematic, kinetic, and temporospatial parameters between Ch-GA and Ad-GA. Chi-square tests were used to assess differences between groups in the number of participants who had undergone orthopaedic or neurosurgery between Ch-GA and Ad-GA, as well as at Ad-GA, including shunted hydrocephalus, joint contractures, spasticity, and additional neurological signs.

Statistical analyses were performed using SPSS, Version 28.0. The significance level was set at *p* < 0.05.

## Results

At Ad-GA, 22/29 participants had maintained independent, non-assisted walking (Group A), 5/29 had transitioned from independent non-assisted walking to walking with an aid (Group B), and 2/29 used a walking aid at both Ch-GA and Ad-GA (Group C) (Table 1).

**Table 1:** Patient characteristics in Group A (maintained non-assisted walking), Group B (changed to walking aid) and Group C (unchanged with walking aid) at gait analysis in childhood (Ch-GA) and in adulthood (Ad-GA). All values shown are mean (SD)

	Group A (*n* = 22)		Group B (*n* = 5)		Group C (*n* = 2)	
	Ch-GA	Ad-GA	Ch-GA	Ad-GA	Ch-GA	Ad-GA
Age (years)	11.4 (3.3)	25.3 (3.7)	12.7 (1.9)	28.2 (3.5)	11.7 (3.8)	27.7 (2.9)
Height (m)	1.39 (0.2)	1.63 (0.1)	1.49 (0.1)	1.61 (0.1)	1.37 (0.1)	1.64 (0.1)
Weight (kg)	40.0 (13.7)	72.5 (15.8)	48.8 (1.9)	69.1 (15.8)	36.2 (11.0)	60.5 (2.1)
Body Mass Index	20.0 (3.4)	27.4 (5.8)	22.1 (3.3)	26.9 (6.4)	19.2 (2.2)	22.7 (2.2)

*n* Number of participants, *Ch-GA* Childhood gait analysis,* Ad-GA* Adult gait analysis, *SD* Standard deviation, *m* Meter, *kg* Kilogram

Functional ambulation was preserved between Ch-GA and Ad-GA in 15/29 (52%) participants. Functional ambulation at Ch-GA and Ad-GA for each participant in Groups A, B, and C, with respect to MFC at Ad-GA, is shown in Table 2.

**Table 2:** Functional ambulation and orthosis used at gait analysis in childhood (Ch-GA) and adulthood (Ad-GA) for each participant in Group A (maintained non-assisted walking), Group B (changed to walking aid) and Group C (unchanged with walking aid) with respect to muscle function class (MFC)

Group/MFC	Functional ambulation		Orthoses	
	Ch-GA	Ad-GA	Ch-GA	Ad-GA
Group A (*n* = 22)				
MFC I				
1	Ca	Ca	FO	FO
2	Ca	Ca	SMO L	SMO L
3	Ca	Ca	AFO-H	FO
4	Ca	Ca	AFO-H	FO
5	Ca	Ca	FO	FO
MFC II				
6	Ca	Ha	AFO-O	AFO-S
7	Ca	Ca	AFO-S	AFO-S
8	Ca	Ha	AFO-C	AFO-C
9	Ca	Ha	AFO-C	AFO-C
10	Ca	Ha	AFO-O	AFO-S
11	Ca	Ha	AFO-C	KAFO-F-C
12	Ca	Ca	AFO-C	AFO-C
13	Ca	Ha	KAFO-F-C	KAFO-F-C
14	Ca	Ha	Shoes	Shoes
15	Ca	Ca	SMO	Shoes
MFC III				
16	Ha	Ha	AFO-O	KAFO-F-C
17	Ca	Ha	KAFO-F-O	AFO-S
18	Ca	Ha	AFO-C	AFO-C L / KAFO-F-C R
19	Ca	Ha	KAFO-F-C	KAFO-F-C
20	Ha	Ha	KAFO-F-O	KAFO-F-S
21	Ha	Ha	KAFO-F-C	AFO-C
22	Ha	Ha	KAFO-F-O	AFO-C
Group B (*n* = 5)				
MFC III				
23	Ha	Ha ^c)^	KAFO-F-O	KAFO-F-H L / AFO-H R
24	Ha	N-f ^c)^	KAFO-F-O	KAFO-F-H
25	Ha	N-f ^b)^	AFO-O	AFO-O
26	Ha	Ha ^b)^	KAFO-F-H	KAFO-F-H
27	Ha	N-f ^c)^	KAFO-F-O	AFO-O
Group C (*n* = 2)				
MFC II				
28	Ha ^a)^	N-f ^c)^	KAFO-F-O	AFO-S
MFC III				
29	Ha ^b)^	Ha ^d)^	KAFO-L-O	KAFO-L-O

*Ca* Community ambulation, *Ha* Household ambulation, *N-f* Non-functional ambulation, *FO* Foot orthosis, *SMO* Supramalleolar orthosis, *L* Left, *R* Right, *AFO-H* Ankle-foot orthosis hinged ankle joint with restricted range of motion, *AFO-O* Ankle-foot orthosis with overlap ankle joint, *AFO-S* Ankle-foot orthosis solid, *AFO-C* Ankle-foot orthosis with carbon fiber spring ankle joint, *KAFO-F-C* Knee-ankle-foot orthosis free articulating knee joint and carbon fiber spring ankle joint, *KAFO-F-O* Knee-ankle-foot orthosis free articulating knee joint and overlap ankle joint, *KAFO-L-O* Knee-ankle-foot orthosis with locked knee joint and overlap ankle joint, *KAFO-F-S* Knee-ankle-foot orthosis with free articulating knee joint and solid ankle joint, *KAFO-O-H* Knee-ankle-foot orthosis with free articulating knee joint and hinged ankle joint with restricted range of motion,

^(a)^ walked with canes

^(b)^ walked with anterior rollator

^(c)^ walked with forearm crutches

^(d)^ walked with anterior rollator with forearm support

Orthoses were used by 28/29 participants at Ch-GA and by 27/29 at Ad-GA (Table 2). Orthosis types, materials and components are detailed in the Supplementary Material.

At Ad-GA, 17/22 participants in Group A and all participants in Group B (5/5) had shunted hydrocephalus (*p* = 0.238), as did both participants in Group C.

Between Ch-GA and Ad-GA, 13/22 participants in Group A and 1/5 participant in Group B had undergone orthopaedic surgery (*p* = 0.163), and 7/22 participants in Group A and 3/5 participants in Group B had undergone neurosurgery (*p* = 0.239). Both participants in Group C had undergone orthopaedic surgery, and one had undergone neurosurgery between Ch-GA and Ad-GA (Table 3). At Ad-GA, there were no differences between Groups A and B in the prevalence of joint contractures, lower-limb spasticity, or additional neurological signs (Table 3).

**Table 3:** Joint contractures, spasticity and additional neurological signs at Ad-GA. Type of performed orthopaedic surgery and number of procedures between Ch-GA and Ad-GA and performed neurosurgery and number of procedures between Ch-GA and Ad-GA, in Group A (maintained non-assisted walking), Group B (changed to walking aid) and Group C (unchanged with walking aid)

	Group A (*n* = 22)		Group B (*n* = 5)		Group C (*n* = 2)		*P*-value^2^
At Ad-GA							
Joint contractures (unilateral and bilateral)	n^1^	Degrees median [min, max]	n^1^	Degrees median [min, max)	n^1^	Degrees median [min, max)	
- hip flexion	4	5 [5, 15]	3	20 [10, 30]	2	25 [15, 35]	ns^3^
- knee flexion	10	10 [5, 30]	3	25 [20, 25]	2	27 [15, 40]	ns^3^
- plantarflexion	4	10 [5, 20]	0	0	0	0	ns^3^
Spasticity in lower limbs^1^	9		3		2		ns^3^
Additional neurological signs^1^	3		1		2		ns^3^
Between Ch-GA - Ad-GA							
Orthopaedic surgery	n^1^	n procedures	n^1^	n procedures	n^1^	n procedures	
Spine	1	1	1	1	1	1	-
Pelvis, hip (uni, bi)	4	4	0	-	2	4	-
Knee (uni, bi)	2	3	0	-	0	-	-
Tibia (uni, bi)	0	-	0	-	0	-	-
Foot, ankle (uni, bi)	10	14	1	1	1	1	-
Neurosurgery^1^	7	10	3	5	1	1	-

^1^number of participants who had contractures, had undergone orthopaedic surgery and neurosurgery

^2^Group C was not included in the statistical analysis

^3^Statistical calculations were based on the number of participants with unilateral and bilateral joint contractures

At Ad-GA, 3/22 participants in Group A reported pain on the examination day: one in MFC II (right hip, VAS 70) and two in MFC III (back, VAS 15 and VAS 60, respectively).

No participants in Groups B or C reported pain on the examination day.

### Gait analysis

Three-dimensional gait parameters were analysed for participants who walked without any external assistance at both Ch-GA and Ad-GA (Group A). GDI and temporospatial parameters were analysed in Group B. Results from the two participants who used a walking aid at both gait analysis examinations are presented individually (Group C). 

#### Group A

Trunk kinematics from Ch-GA was not available in four participants (two in MFC I and two in MFC II). Kinetic data was missing in one participant at Ad-GA in MFC III. There was no difference in GDI between left and right sides at Ch-GA: median [min, max] 61.5 [49.2, 87.9] vs. 61.5 [45.8, 87.4] (*p* = 0.833), nor at Ad-GA: 64.7 [45.6, 87.4] vs. 60.4 [48.3, 89.5] (*p* = 0.223). Results of both legs for GDI, kinematics, joint moments, contribution (as percent) to total work in hip, knee and ankle, and temporospatial parameters in MFC I, MFC II and MFC III at Ch-GA and Ad-GA are presented in Table 4.

**Table 4:** Gait Deviation Index, kinematics, joint kinetics, and non-dimensional time and distance parameters in Group A (maintained non-assisted walking), according to MFC (muscle function classes) at childhood gait analysis (Ch-GA) and at adult gait analysis (Ad-GA). All values shown are median [minimum, maximum]

Group A (*n* = 22)	MFC 1 (*n* = 5)			MFC II (*n* = 10)			MFC III (*n* = 7)		
Median [min, max]	Ch-GA	Ad-GA	*P-value*	Ch-GA	Ad-GA	*P-value*	Ch-GA	Ad-GA	*P-value*
Gait Deviation Index (GDI)	65.6 [51.6, 77.0]	81.3 [68.9, 88.4]	0.080	66.7 [49.7, 87.6]	63.1 [53.1, 87.3]	0.878	54.9 [52.8, 65.6]	54.3 [46.9, 62.5]	0.735
Kinematics (°)									
Average trunk tilt anterior ^a)^	1.2 [1.1, 2.7]	-0.5 [-2.5, 6.3]	0.593	-0.4 [-15.2, 5.7]	-0.9 [-10.4, 3.7]	0.779	5.3 [-8.4, 10.5]	5.1 [-8.1, 8.7]	0.499
Trunk lateral sway range	3.8 [3.7, 8.1]	4.9 [1.9, 6.8]	1.000	16.3 [7.0, 28.8]	19.0 [7.0, 28.1]	0.575	40.1 [24.0, 56.1]	43.4 [20.8, 55.0]	0.237
Trunk rotation range	6.1 [5.2, 8.5]	9.3 [7.1, 17.3]	0.109	12.6 [8.7, 20.2]	12.2 [6.1, 18.1]	0.208	21.3 [11.8, 36.6]	17.4 [12.8, 34.6]	0.398
Average pelvic tilt	16.9 [10.7, 27.1]	18.0 [12.6, 19.0]	0.500	22.7 [6.0, 32.7]	23.7 [10.8, 26.1]	0.575	26.7 [18.3, 31.4]	24.2 [15.0, 30.8]	0.612
Pelvic obliquity max in swing	1.0 [0.3, 2.4]	1.1 [-0.9, 2.2]	0.225	4.8 [0.8, 14.0]	4.1 [-0.3, 11.2]	0.285	9.9 [4.2, 14.3]	9.0 [6.3, 14.2]	1.000
Pelvic rotation range	16.0 [12.3, 19.1]	14.6 [5.6, 21.2]	0.893	26.0 [7.4, 48.8]	23.7 [5.4, 50.7]	0.169	40.3 [28.1, 69.3]	43.4 [33.5, 66.8]	0.310
Minimum hip flexion ^b)^	-1.6 [-7.1, 10.1]	-3.2 [-7.2, 2.3]	0.345	3.7 [-18.7, 16.1]	7.3 [-9.6, 19.4]	0.114	0.9 [-12.2, 7.1]	10.6 [-7.9, 28.8]	0.128
Hip flexion-extension range	53.2 [46.6, 60.5]	47.6 [45.0, 53.1]	0.225	48.0 [39.0, 72.7]	47.9 [32.3, 54.4]	**0.047**	54.7 [30.5, 60.6]	39.8 [18.9, 57.3]	**0.028**
Knee flexion max in swing	61.0 [50.7, 74.7]	60.4 [52.2, 61.9]	0.345	58.4 [40.6, 76.0]	55.5 [43.4, 75.2]	0.139	54.0 [40.2, 64.6]	50.5 [33.6, 65.5]	0.499
Knee flexion min in midstance ^c)^	12.6 [-3.6, 24.2]	2.9 [-1.2, 18.8]	0.138	13.4 [-2.7, 39.1]	15.3 [-6.5, 43.8]	0.575	4.0 [-7.3, 12.6]	16.2 [-9.7, 30.7]	0.128
Average foot progression in stance ^d)^	4.1 [-9.1, 23.0]	-5.2 [-13.5, 11.0]	0.080	-8.5 [-24.0, 2.8]	-10.2 [-20.9, 14.2]	0.575	-5.8 [-19.9, 8.6]	-5.0 [-22.4, 13.4]	0.866
Internal joint moments (Nm/kg)									
Max hip extension	0.86 [0.44, 1.44]	1.06 [0.48, 1.81]	0.686	0.89 [0.54, 1.58]	1.11 [0.68, 1.76]	0.093	0.83 [0.32, 1.53]	0.88 [0.47, 1.51]	**0.046**
Average hip abduction in stance ^e)^	0.26 [0.06, 0.49]	0.45 [0.41, 0.54]	**0.043**	0.16 [-0.29, 0.45]	0.22 [-0.28, 0.51]	0.959	-0.25 [-0.46, 0.11]	-0.33 [-0.40, 0.15]	0.345
Knee extension min in mid-stance) ^f)^	-0.05 [-0.30, 0.19]	-0.24 [-0.32, 0.16]	0.138	-0.09 [-0.45, 0.13]	0.07 [-0.35, 0.37]	**0.047**	-0.24 [-0.49, -0.01]	-0.08 [-0.86, 0.36]	0.345
Knee valgus average in stance ^g)^	0.03 [0.03, 0.34]	0.26 [0.17, 0.31]	0.080	0.01 [-0.30, 0.28]	0.13 [-0.37, 0.28]	0.646	-0.07 [-0.35, 0.08]	-0.22 [-0.35, 0.03]	**0.028**
Max ankle dorsiflexion	-0.19 [-0.79, -0.08]	-0.16 [-0.27, -0.06]	0.500	-0.24 [-0.71, -0.03]	-0.23 [-0.32, -0.14]	0.799	-0.24 [-0.38, -0.12]	-0.17 [-0.26, -0.14]	0.249
Max ankle plantarflexion	0.82 [0.25, 1.17]	0.77 [0.33, 1.15]	0.893	1.14 [0.65, 1.47]	0.94 [0.71, 1.14]	**0.022**	1.10 [0.76, 1.49]	1.22 [0.75, 1.33]	0.917
Contribution of total positive work (%)									
Hip	42.6 [28.5, 56.2]	46.3 [44.2, 55.9]	0.225	41.2 [30.9, 57.3]	52.6 [37.5, 73.2]	**0.013**	34.8 [27.5, 59.6]	39.7 [31.4, 57.9]	0.345
Knee	33.6 [27.1, 47.2]	31.1 [14.8, 45.7]	0.500	31.7 [19.9, 48.1]	30.6 [15.9, 44,7]	0.386	40.5 [29.2, 53.8]	46.9 [22.6, 59.6]	0.249
Ankle	23.2 [12.3, 31.0]	21.4 [10.2, 29.3]	0.500	23.3 [13.5, 43.3]	14.9 [9.2, 33.4]	**0.047**	17.9 [10.5, 30.3]	11.5 [9.1, 20.0]	0.075
Non-dimensional time and distance parameters									
N stride length	1.44 [1.33, 1.74]	1.43 [1.17, 1.63]	**0.043**	1.54 [1.17, 1.96]	1.51 [0.93, 1.73]	0.093	1.61 [0.79, 1.97]	1.20 [0.64, 1.63]	**0.043**
N step length	0.72 [0.66, 0.87]	0.71 [0.59, 0.82]	**0.043**	0.77 [0.57, 0.98]	0.75 [0.47, 0.87]	0.059	0.82 [0.41, 0.99]	0.59 [0.34, 0.81]	**0.043**
N walking speed	0.48 [0.35, 0.50]	0.39 [0.29, 0.47]	**0.043**	0.40 [0.33, 0.53]	0.37 [0.23, 0.44]	**0.022**	0.43 [0.20, 0.49]	0.32 [0.13, 0.41]	**0.043**

*MFC* Muscle function class, *max* Maximum, *min* Minimum, *N* Non-dimensional

Significant differences are indicated with bolded *p*-values

^(a)^ (–) = posterior

^(b)^ (–) = extension

^(c)^ (–) = extension

^(d)^ (–) = internal

^(e)^ (–) = adduction moment

^(f)^ (–) = flexion moment

^(g)^ (–) = varus moment

**Fig. 1:**
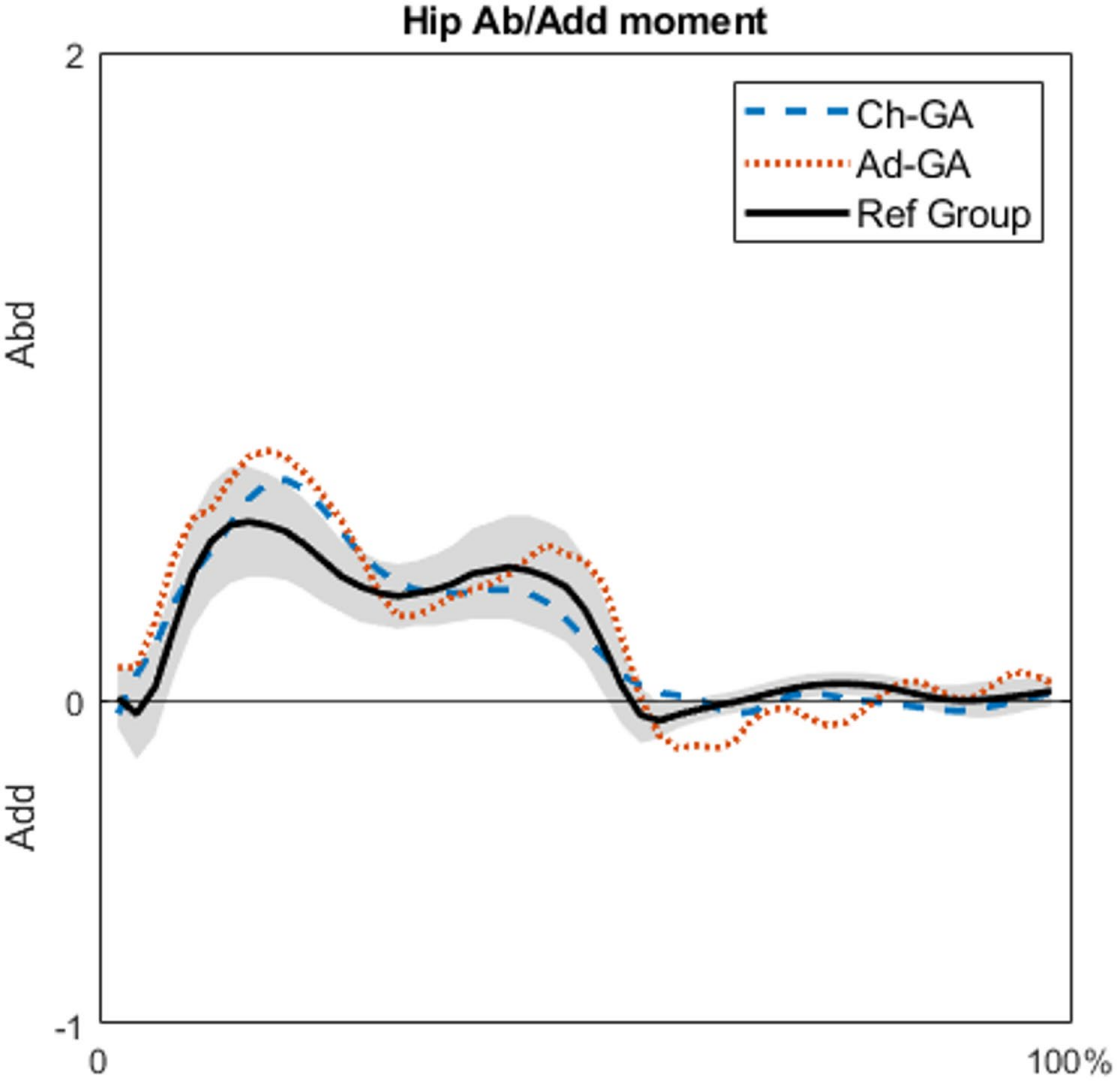
Illustration of internal hip abduction/adduction moment (Nm/kg) as a mean of three gait cycles in one participant in Group A in MFC I at gait analysis in childhood (Ch-GA) and at adulthood (Ad-GA). This participant showed the greatest difference in internal hip abduction/adduction moment between Ch-GA and At-GA. The reference group (Ref group) is represented by the mean ± 1 SD of 37 typically developed children of the gait laboratory

In MFC I, at Ad-GA vs. Ch-GA, average hip abduction moment during stance was greater (*p* = 0.043) (Fig. 1), N stride length was shorter (*p* = 0.043), N step length was shorter (*p* = 0.043), and N walking speed was slower (*p* = 0.022).

**Fig. 2:**
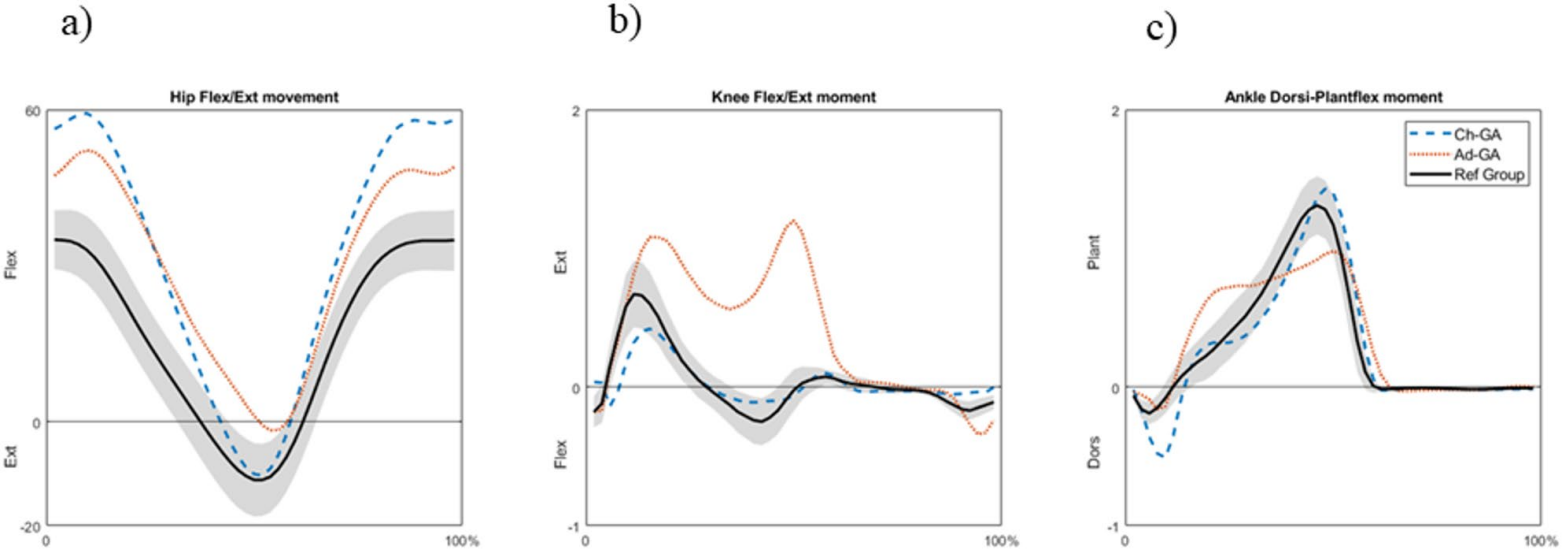
**A** Illustration of knee flexion/extension movement (degrees) as a mean of three gait cycles in one participant in Group A in MFC II at gait analysis in childhood (Ch-GA) and at adulthood (Ad-GA). This participant showed the greatest difference in knee flexion/extension movement between Ch-GA and At-GA. The reference group (Ref group) is represented by the mean ± 1 SD of 37 typically developed children of the gait laboratory. **B** Illustration of internal knee flexion/extension moment (Nm/kg) as a mean of three gait cycles in one participant in Group A in MFC II at gait analysis in childhood (Ch-GA) and at adulthood (Ad-GA). This participant showed the greatest difference in internal knee flexion/extension moment between Ch-GA and Ad-GA. The reference group (Ref group) is represented by the mean ± 1 SD of 37 typically developed children of the gait laboratory. **C **Illustration of internal dorsi flexion/plantarflexion moment (Nm/kg) as a mean of three gait cycles in one participant in Group A in MFC II at gait analysis in childhood (Ch-GA) and at adulthood (Ad-GA). This participant showed the greatest difference in internal dorsi flexion/plantarflexion moment between Ch-GA and Ad-GA. The reference group (Ref group) is represented by the mean ± 1 SD of 37 typically developed children of the gait laboratory

**Fig. 3:**
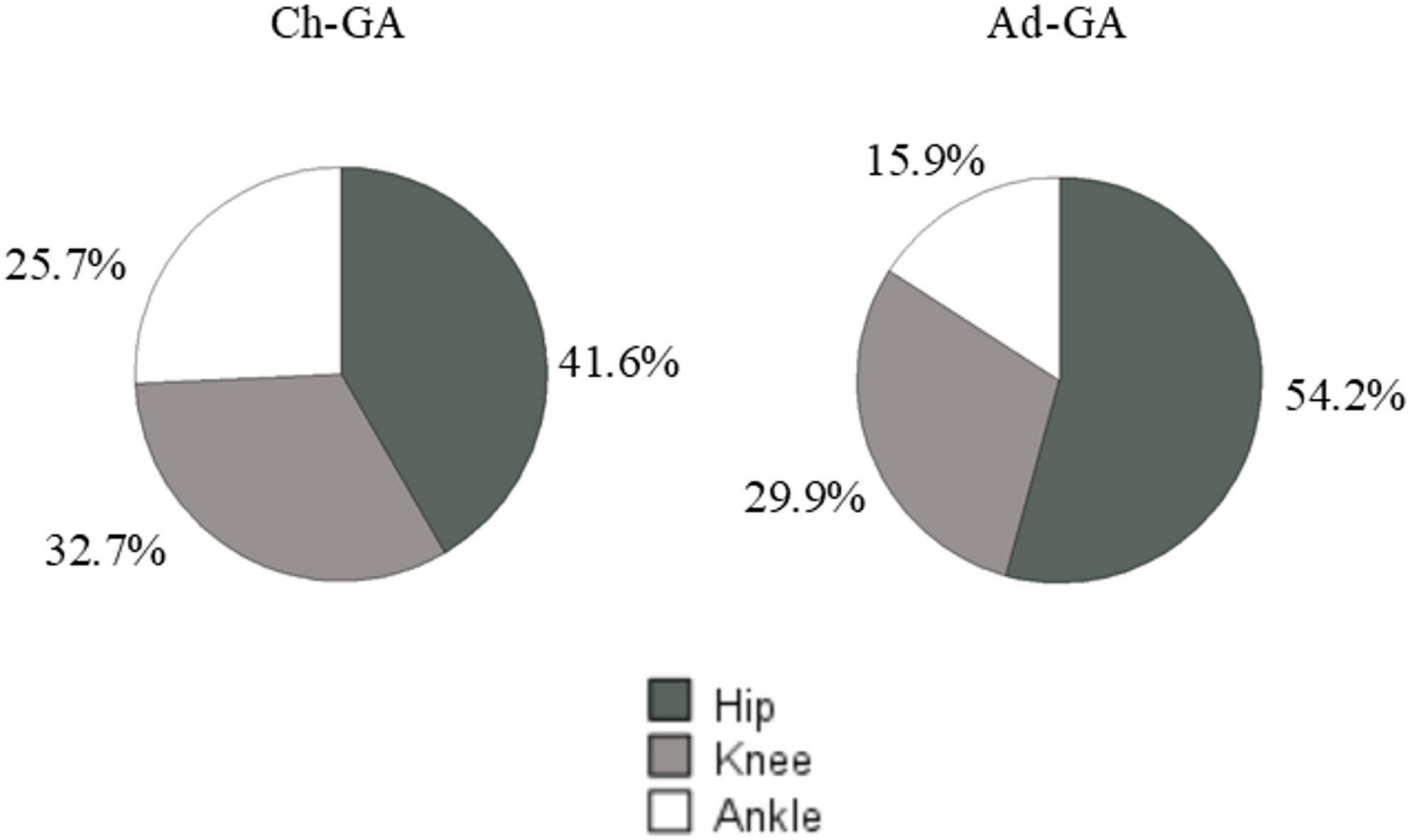
Illustration of mean changes in contribution to total positive work in percent (%) between gait analysis at childhood (Ch-GA) and at adulthood (Ad-GA) in hip, knee and ankle in Group A in Muscle Function Class II. There were significant differences in hip (p=0.013) and in ankle joint (p=0.047)

In MFC II, at Ad-GA vs. Ch-GA, hip flexion-extension range decreased (*p* = 0.047) (Fig. 2a), knee joint moment in mid-stance in sagittal plane changed directions from an knee flexion moment to an knee extension moment (*p* = 0.047) (Fig. 2b), and maximum plantarflexion moment decreased (*p* = 0.022) (Fig. 2c). At Ad-GA, contributions to total positive work in the lower extremities increased at the hip (*p* = 0.013) and decreased at the ankle (*p* = 0.047) (Fig. 3) since Ch-GA. At Ad-GA versus Ch-GA, N walking speed was slower (*p* = 0.022).

**Fig. 4:**
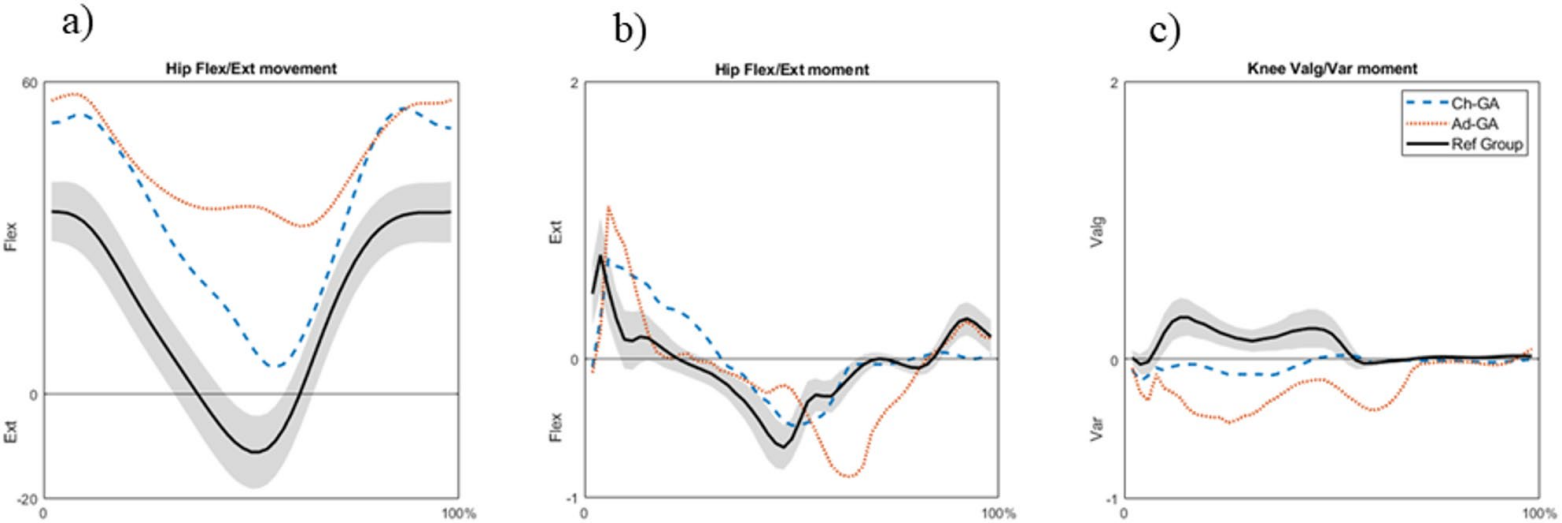
**A** Illustration of hip flexion/extension movement (degrees) as a mean of three gait cycles in one participant in Group A in Muscle Function Class III (MFC III) at gait analysis in childhood (Ch-GA) and at adulthood (Ad-GA). This participant showed the greatest difference in knee flexion/extension movement between Ch-GA and Ad-GA. The reference group (Ref group) is represented by the mean ± 1 SD of 37 typically developed children of the gait laboratory. **B **Illustration of internal hip flexion/extension moment (Nm/kg) as a mean of three gait cycles in one participant in Group A in Muscle Function Class III (MFC III) at gait analysis in childhood (Ch-GA) and at adulthood (Ad-GA). This participant showed the greatest difference in internal hip flexion/extension moment between Ch-GA and Ad-GA. The reference group (Ref group) is represented by the mean ± 1 SD of 37 typically developed children of the gait laboratory. **C** Illustration of internal knee varus/valgus moment (Nm/kg) as a mean of three gait cycles in Group in Muscle Function Class (MFC III) at gait analysis at childhood (Ch-GA) and at adulthood (Ad-GA). This participant showed the greatest difference in internal knee varus/valgus moment between Ch-GA and Ad-GA. The reference group (Ref Group) is represented by the mean ± 1 SD of 37 typically developed children of the gait laboratory

In MFC III, at Ad-GA vs. Ch-GA, hip flexion-extension range decreased (*p* = 0.028) (Fig. 4a), maximum hip extension moment in sagittal plane increased (*p* = 0.046) (Fig. 4b), and average knee varus moment in stance increased (*p* = 0.028) (Fig. 4c). At Ad-GA versus Ch-GA, N stride length was shorter (*p* = 0.043), N step length was shorter (*p* = 0.043), and N walking speed was slower (*p* = 0.043).

GDI did not differ in any of the MFC groups in Group A between Ch-GA and Ad-GA. 

#### Group B

The GDI at Ad-GA was lower at the left side than at the right side: 52.8 [39.4, 66.2] vs. 57.7 [35.0, 75.2] (*p* = 0.043), but were not significantly different at Ch-GA; 60.9 [49.2, 63.8] vs. 63.7 [56.0, 75.9] (*p* = 0.893). At Ad-GA vs. Ch-GA, N stride length was shorter (*p* = 0.043), N step length was shorter (*p* = 0.043), and N walking speed was slower (*p* = 0.043) (Table 5).

**Table 5:** Gait Deviation Index (GDI) and non-dimensional (N) time and distance parameters from gait analysis in childhood (Ch-GA) and adulthood (Ad-GA) in Group B (changed to walking aid). All values are shown as median [min, max]

Group B (*n* = 5)	Ch-GA	Ad-GA	*p*-value
GDI	62.3 [53.5, 69.9]	58.5 [46.6, 65.2]	0.138
N stride length	1.39 [1.24, 1.59]	1.11 [0.96, 1.35]	**0.043**
N step length	0.70 [0.61, 0.78]	0.54 [0.49, 0.68]	**0.043**
N walking speed	0.35 [0.29, 0.36]	0.25 [0.15, 0.32]	**0.043**

Bolded *p*-values indicate significant differences

#### Group C

In one of the two participants, the GDI of both legs was 65.4 and 62.7, N stride length was 1.00 and 1.06, N step length was 0.50 and 0.52, and N walking speed was 0.17 and 0.21 at Ad-GA and Ch-GA, respectively. In the other participant, the GDI of both legs was 44.5 and 46.9, N stride length was 1.23 and 1.00, N step length was 0.61 and 0.56, and N walking speed was 0.25 and 0.16 at Ad-GA and Ch-GA, respectively.

## Discussion

In this retrospective study, in persons with MMC, we aimed to explore whether gait patterns as an adult remained unchanged from that in childhood. This hypothesis was based particularly on the fact that the orthotic management in the same cohort had changed only marginally from childhood to adulthood [2]. The most interesting finding is that 22 (Group A) of the original 27 participants who walked independently as children had maintained independent, non-assisted walking with only few changes in kinematic and kinetic gait variables, although 10 of the 18 community ambulators had shifted to household ambulation.

Not in accordance with our hypothesis, however, five (Group B) of the original 27 participants who walked independently in childhood had changed to using walking aid as an adult, of whom three changed from household ambulation to non-functional ambulation.

In Group A we analyzed 3D gait patterns according to three muscle function groups. In MFC I, muscle paresis is predominantly located in the intrinsic foot muscles but may also include slight weakness of the toe flexors and plantarflexors [24]. In this group, an improvement in hip abduction moment during stance was observed. This improvement may not be directly attributable to the level of paresis may instead be associated with the extensive orthopaedic surgery involving the hip and knee undergone by one of the five participants.

Moreover, in MFC I stride and step length was shorter while walking speed was slower between Ch-GA and Ad-GA. These differences were small and therefore cannot be considered clinically relevant. Two of the five participants had changed orthoses from AFOs to FOs between Ch-GA and Ad-GA, at remaining similar stride and step length.

In MFC II, characteristic plantarflexor paresis and hip extensor weakness are commonly observed and may contribute to increased hip flexion during walking [8]. In MMC II, the slight decrease in hip flexion–extension range found at Ad-GA can be regarded as a positive development, with reduced initial hip flexion and maintained hip extension to a neutral level during mid-stance, which was confirmed by higher total positive work at the hip. The shift at Ch-GA from a normal knee flexion moment in mid-stance, defined here as 20–50% of the gait cycle, to a pronounced knee extension moment at Ad-GA is considered to reflect knee flexion contractures of 5–30 degrees, present in six of the ten participants. These contractures may in turn have been influenced by spasticity. To counteract tibial advancement caused by plantarflexor weakness and to avoid transverse rotational forces at the knee [24], orthoses with restricted ankle dorsiflexion (AFOs or KAFO-Fs) were used by eight participants at both Ch-GA and Ad-GA. One participant used supramalleolar orthosis bilaterally during childhood but had stopped using them in adulthood. The presence of knee flexion contractures resulted in an increased knee extension moment and may also be reflected in the plantarflexion moment during mid-stance, possibly interacting with the mechanical behaviour of the carbon spring [11]. Since all five carbon fibre spring orthoses users had the same type of orthoses at both gait analyses, the variability in plantarflexor moment and the suboptimal roll-over motion—confirmed by reduced total positive work at the ankle—are believed to be attributable to the knee contractures. Eight of the ten participants had undergone lower-limb orthopaedic surgery, and two had undergone untethering of the spinal cord, circumstances that also may reflect the slower walking speed in adulthood. At Ad-GA, one participant reported right hip pain on the day of examination, which may have influenced gait; however, this could not be confirmed by the measured parameters.

In MFC III, the hip flexion–extension movement range was approximately 15 degrees smaller at Ad-GA than at Ch-GA, with the reduction occurring primarily during mid-stance.

This may be partly explained by hip flexion contractures of 5–15 degrees, which were present in four of the seven participants at the adult examination. The increased hip flexion at initial contact also appears to correspond to the increase in internal hip extension moment. One participant had undergone unilateral hip and femoral varus osteotomy surgery between the two gait analyses and in four participants before Ch-GA. No participants presented with hip dislocation. In MFC III, greater knee varus moments during stance were also observed in adulthood than in childhood. Since individuals with MFC III typically present with lateral trunk pendulum motion, they frequently receive KAFO-Fs to counteract knee valgus position resulting from hip abductor weakness [7, 8] while not restricting the knee flexion/extension.

In the present group of seven participants, five used bilateral KAFO-Fs in childhood, and three used bilateral and one unilateral KAFO-Fs in adulthood. The increased knee varus moment seen at Ad-GA may therefore be partly attributable to reduced frontal-plane control from orthotic management, but possibly also to higher body weight. Participants in MFC III additionally presented with shorter stride and step lengths and slower walking speed, despite the use of carbon fibre spring components at the ankle in five of seven participants. These components have previously been shown to improve certain gait parameters in children with MMC [11]. However, reductions in temporospatial parameters in adulthood may coincide with several factors, including higher body weight without corresponding increases in muscle strength relative to the degree of paresis, as well as the need to reduce step length due to hip extensor weakness, which may result in compensatory increases in trunk movements [8].

Additionally, people with MMC are very likely to have impaired proprioception in joints that are surrounded by paralyzed muscles, which can affect gait. However, a study in isolated knee joint position sense showed no impaired proprioception which is thought to be associated with full innervation in the quadriceps muscles, although this strength might not be able to be fully utilized in standing due to the calf muscle weakness that occurs already at a low neurological level [34]. Children with MMC also perceived somatosensory stimuli less clearly than typically developing children in all tests, more noticeable at higher lesion levels. Therefore, by documenting a child’s tactile sensation, the risk of skin damage and pressure ulcers can be avoided, especially if orthotics are used [35]. Early monitoring of walking speed and weight management during follow-up and designing rehabilitation programs for MMC that encourage achieving and maintaining sustainable gait performance have been suggested [14, 15]. Back pain, which has been reported among adults with MMC in this cohort [26], may also have influenced gait in the present study; three participants in Group A reported pain after completing the gait analysis at Ad-GA. Among the 22 participants in Group A, the GDI did not change significantly from childhood to adulthood that may likely be due to the large magnitude of difference in all three groups. In MFC 1, we noticed an improvement in the GDI of more than 10 points that may reflect the increased hip abduction moment at Ad-GA. In MFC II and MFC III the GDI values remained stable over time.

In Group B, who walked with a walking aid at Ad-GA, the mean GDI had slightly decreased from childhood to adulthood, but not significantly. At both examinations, however, the GDI was approximately half of the normal value [32], and comparable to the GDI in MFC I and II within Group A. All five participants in Group B had household ambulation at Ch-GA, but three no longer walked as part of everyday function at Ad-GA. Median hip and knee flexion contractures were 5 and 10 degrees, respectively, in MFC III in Group A, compared with 20 and 25 degrees in Group B, whose participants also belonged to MFC III.

Of the five participants in Group B who used walking aids, two used forearm crutches to assist forward progression with their upper extremities (5) and three used KAFO-Fs without a knee-lock mechanism. Due to the altered biomechanical conditions in those who no longer walked without assistance in adulthood, no 3D gait comparison was performed. To illustrate the low GDI values observed in Group B, one participant with a GDI of 35 at Ad-GA demonstrated pelvic obliquity of approximately 15 degrees and anterior pelvic tilt of approximately 20 degrees during both stance and swing phases. This participant also exhibited a shorter step length at Ad-GA (0.39 m) than at Ch-GA (0.49 m), and slower walking velocity (0.49 m/s versus 0.91 m/s). In Group B, three of the five participants presented with lower-limb spasticity and had undergone neurosurgical interventions.

In Group C, one participant belonged to MFC II and one to MFC III. The participant with MFC III was still a household ambulator and was the only individual in the entire cohort who used locked KAFOs at both gait analysis examinations. In contrast, the participant with the lower neurological lesion (MFC II) demonstrated non-functional ambulation in adulthood and had a lower GDI. Both participants exhibited extensive lower-limb spasticity and marked spatial fear when standing upright. In addition, both had undergone extensive surgical procedures between the two gait analysis examinations.

In the present study, no participant used an orthosis that included hip-joint coverage [13]. However, four participants had used a hip–knee–ankle–foot orthosis (HKAFO) during early childhood in accordance with the orthotic program, with the aim of stabilizing posture during standing. Once stable hip joints were confirmed, the children transitioned to KAFO-Fs when beginning to walk. In cases of persistent hip-joint instability, a reciprocating gait orthosis would have been indicated [20].

Many authors agree on the importance of assessing the key muscle groups required to achieve an upright posture in the sagittal and frontal planes [24, 36–38], as these muscle functions serve as an important guideline for predicting walking ability. The designation of muscle function classes differentiates between critical stages of increasing muscle paresis in MMC, including paresis of the plantarflexors, hip extensors, hip abductors, knee extensors, hip adductors and hip flexors. Nevertheless, the results from the present study indicate—consistent with previous findings—that a patient’s walking potential may be influenced by additional factors beyond the neurological lesion level alone [16–18]. For example, some children may not achieve the expected level of walking ability based on their muscle function and therefore require more extensive orthotic support due to balance disturbances or spasticity, as observed in one participant in Group C who required locked KAFOs despite good knee extension strength. Considering additional neurological factors as well as other influencing conditions is therefore an important aspect in planning interventions aimed at achieving upright posture and ambulation in individuals with MMC [20].

We acknowledge several limitations in this study. Placing markers on orthoses presents challenges. However, all markers were placed by the same examiner at both the childhood and adult gait analyses, minimizing the risk for inter-examiner variability. Another limitation is the small sample size, which was further reduced when subdividing participants into muscle function classes within Group A, and especially in Group C, which comprised only two participants. Statistical analyses based on such small groups as in the present study may not provide clinically meaningful insights and should therefore be interpreted with caution. Such subdivision was nevertheless essential, as orthotic needs differ substantially between muscle function groups. GDI, furthermore, is computed on the lower limb kinematics, and does not include trunk movements. It may therefore not fully represent the typical gait pattern in MMC, particularly in MFC III, which is characterized by large deviations in trunk movement in both the frontal and transverse planes [8]. The largest difference in GDI between Ch-GA and Ad-GA was observed, although not significant in MFC I. MFC III had the lowest GDI of all groups, which can be expected due to the extent of muscle paresis and more pelvic obliquity compared to MFC I and MFC II: Nonetheless, we accepted using the GDI also for Group B walking with an assistive device as an adult because the influence of trunk movements had only taken place in childhood. However, comparing kinematic and kinetic variables with or without walking aid cannot generally be considered a valid method, being the reason why we did not present 3D results for Group B.

All participants in the present study had access to a comprehensive treatment program that integrated orthotic management, with timing of orthotic intervention, and physiotherapy. 

However, the specific treatments and interventions the participants received during the period between the two gait analyses could not be fully controlled. Despite this, the results may still reflect a thoughtfully structured and continuous rehabilitation approach aimed at supporting ambulation from childhood into adulthood in individuals with MMC.

## Conclusion

A total of 29 participants with MMC were included in the study, of whom 22 (Group A) had maintained non-assisted, independent walking from childhood to adulthood with individualized orthotic provision, with a largely unchanged gait pattern. Some gait pattern deterioration had occurred in participants in MFC II and III. Between childhood and adulthood, walking speed decreased in all MFC groups. Functional ambulation decreased over time in some participants (46%) among MFC I to MFC III groups. Five participants (Group B) had begun using a walking aid since the childhood examination, in whom temporospatial parameters had decreased. Functional ambulation declined in several participants. The two individuals in Group C, who used a walker at both examinations, have largely unchanged gait and temporospatial parameters. Partially in line with our hypothesis, gait patterns remained reasonably stable from childhood to adulthood, supported by adequate and continuous rehabilitation measures. The results also reflect gait and ambulation changes that can be expected during growth in individuals with MMC. 

## Supplementary Information

Supplementary Material 1.

## Data Availability

The datasets used from the current study are available from the corresponding author on reasonable request.

## Abbreviations

MMC: Myelomeningocele
SB: Spina Bifida
MFC: Muscle function class
Ch-GA: Gait analysis at childhood
Ad-GA: Gait analysis at adulthood
3D: Three dimenisional
Ca: Community ambulation
Ha: Household ambulation
N-f: Non-functional ambulation
SD: Standard deviation
GDI: Gait deviation index
FO: Foot orthosis
AFO: Ankle-foot orthosis
KAFO: Knee-ankle-foot orthosis
KAFO-F: Knee-ankle-foot orthosis, free articulated
KAFO-L: Knee-ankle-foot orthosis, locked
VAS: Visual analog scale
N: Non-dimensional
