# Supplementary material for: Gait in childhood and adulthood in persons with myelomeningocele – a retrospective analysis
Source: BMC Neurol. 2026 Jun 23;26:409. doi: 10.1186/s12883-026-05094-y (PMC13289382; doi:10.1186/s12883-026-05094-y)
Supplement: Supplementary file 1 — Supplementary Material 1. [file 12883_2026_5094_MOESM1_ESM.docx]

Orthoses used in Group A in MFC I, MFC II and in MFC III, in Group B and in Group C at childhood (Ch-GA) and at adulthood (Ad-GA).

**Group A** (n=22)

**MFC I** (n=5)


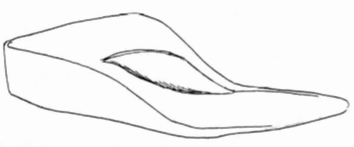

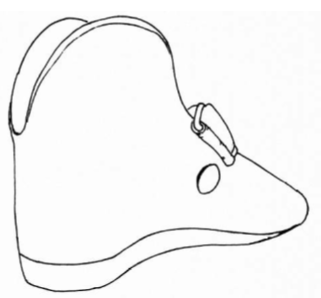

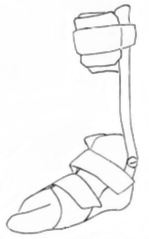


Insole

Supramalleolar orthosis

AFO hinged

with restricted

range of motion

(Ch-GA / Ad-GA) (Ch-GA/ Ad-GA) (Ch-GA)

**MFC II** (n=10)


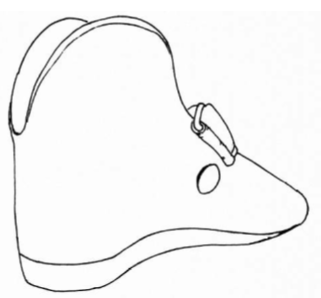

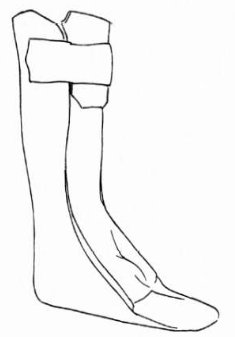

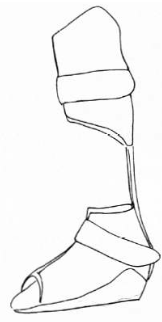

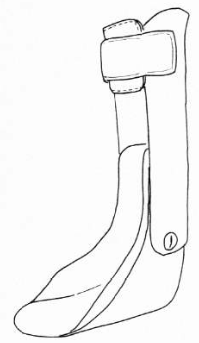

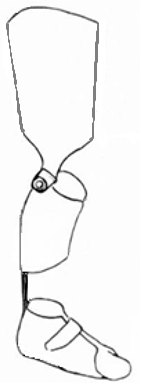


KAFO-F

with carbon

fibre spring

AFO

with carbon

fibre spring

Supramalleolar

orthosis

AFO solid

AFO

with overlap

ankle joint

(Ch-GA) (Ch-GA/ Ad-GA) (Ch-GA) (Ch-GA/ Ad-GA) (Ch-GA/ Ad-GA)

**MFC III** (n=7)


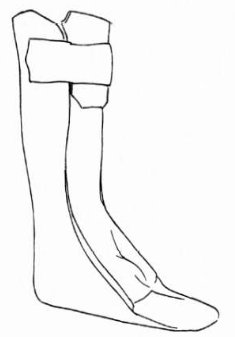

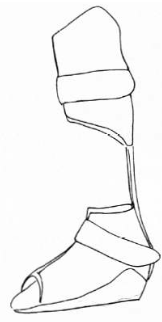

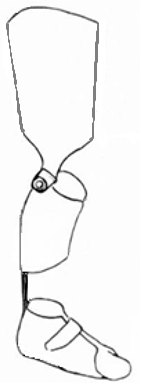

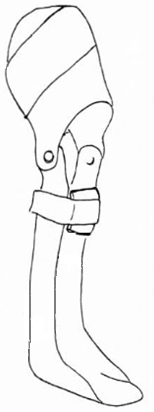

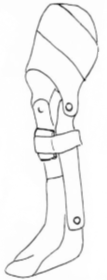

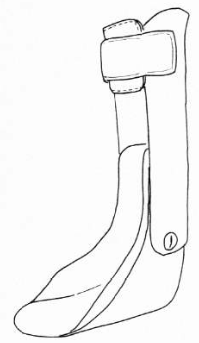


AFO solid

KAFO-F

with carbon

fibre spring

KAFO-F

with solid

ankle

KAFO-F

with overlap

ankle joint

AFO

with carbon

fibre spring

AFO

with overlap

ankle joint

(Ad-GA) (Ch-GA) (Ch-GA/ Ad-GA) (Ch-GA) (Ad-GA) (Ch-GA/ Ad-GA)

**Group B (**n=5)


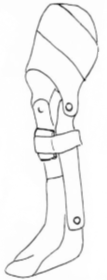

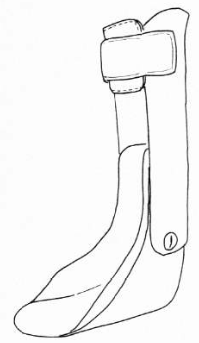

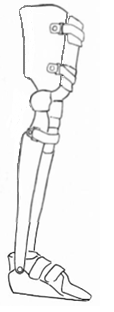


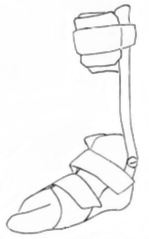


KAFO-F with hinged ankle joint

with restricted range of motion

KAFO-F

with overlap

ankle joint

AFO

with hinged

ankle joint with restricted range of motion

AFO

with overlap

ankle joint

(Ch-GA / Ad-GA) (Ad-GA) (Ch-GA) (Ch-GA / Ad-GA)

**Group C** (n=2)


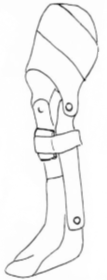

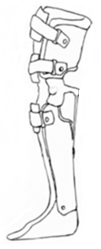


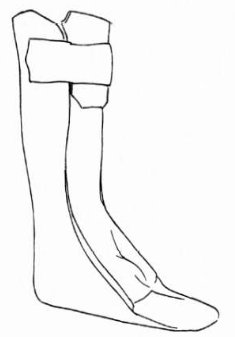


KAFO-F

with overlap

ankle joint

KAFO-L

with overlap

ankle joint

AFO Solid

(Ch-GA) (Ad-GA) (Ch-GA / Ad-GA)

Orthosis types and material in the orthoses at childhood (Ch-GA) and at adulthood (Ad-GA).

| Orthosis type | Material |
| --- | --- |
| **FO** |  |
| Custom fabricated insoles | EVA (ethylene vinyl acetate) |
| SMO | Thermoplastic |
| **AFO** |  |
| Solid (unarticulated) | Shank: composite  Foot: composite merged with thermoplastic from the metatarsal heads |
| Overlap ankle joint | Shank: composite  Foot: thermoplastic |
| Hinged ankle joint, lateral metal  hinge (restricted range of motion) | Shank: composite  Foot: composite merged with thermoplastic from the metatarsal heads |
| Hinged ankle joint, medio-lateral  metal hinge (restricted range of  motion) | Shank: composite  Foot: composite merged with thermoplastic from the metatarsal heads |
| Carbon fibre spring ankle joint | Shank: composite  Foot: composite merged with thermoplastic from the metatarsal heads |
| **KAFO-F** |  |
| Knee: medio-lateral metal hinges  Ankle: solid (unarticulated) | Thigh: composite  Shank: composite  Foot: composite merged with thermoplastic from the metatarsal heads |
| Knee: medio-lateral metal hinges  Ankle: overlap ankle joint | Thigh: thermoplastic  Shank: composite  Foot: thermoplastic |
| Knee: medio-lateral metal hinge  Ankle: carbon fibre spring ankle  joint | Thigh: composite  Shank: composite  Foot: composite merged with thermoplastic from the metatarsal heads |
| Knee: lateral metal hinge  Ankle: carbon fibre spring ankle  joint | Thigh: composite  Shank: composite  Foot: composite merged with thermoplastic from the metatarsal heads |
| Knee: medio-lateral metal hinges  Ankle: hinged ankle joint, medio-  lateral metal hinge (restricted range  of motion) | Thigh: composite  Shank: composite  Foot: composite merged with thermoplastic from the metatarsal heads |
| Knee: lateral metal hinge  Ankle: hinged, lateral metal hinge  (restricted range of motion) | Thigh: composite  Shank: composite  Foot: composite merged with thermoplastic from the metatarsal heads |
| **KAFO-L** |  |
| Knee: medio-lateral metal hinges  with posterior knee locking  mechanism  Ankle: overlap ankle joint | Thigh: composite  Shank: composite  Foot: thermoplastic |

FO; foot orthosis, SMO; supramalleolar orthosis, AFO; ankle-foot orthosis, KAFO-F; knee-ankle-foot orthosis with free articulating knee joint, KAFO-L: knee-ankle-foot orthosis with locked knee joint.
